# Next‐generation sequencing unravels extensive genetic alteration in recurrent ovarian cancer and unique genetic changes in drug‐resistant recurrent ovarian cancer

**DOI:** 10.1002/mgg3.414

**Published:** 2018-05-24

**Authors:** Zhen‐Hua Du, Fang‐Fang Bi, Lei Wang, Qing Yang

**Affiliations:** ^1^ Department of Obstetrics and Gynecology Shengjing Hospital of China Medical University Shenyang China

**Keywords:** gene mutations, liquid biopsy, next‐generation sequencing, ovarian cancer, recurrence, resistance, sensitivity, specificity, survival

## Abstract

**Background:**

By using a high‐throughput sequencing technique, we sought to delineate genetic alterations in recurrent ovarian cancer patients and further compare genetic changes in drug‐resistant and ‐sensitive recurrent ovarian cancer patients. We also sought to study the specificity, sensitivity, and consistency of DNA biomarkers in liquid biopsy specimens and ovarian cancer tissue DNA.

**Methods:**

Tumor tissue specimens and blood samples were obtained from pathologically proven recurrent ovarian cancer patients. Genomic DNA was extracted from tumor tissues, blood cells, ascites, and urine samples. The DNA Library was constructed and sequencing was performed using the Illumina HiSeq 4000 high‐throughput sequencing platform. Bioinformatic analysis was done using the Torrent Suite software.

**Results:**

Ten patients with pathologically proven drug‐resistant recurrent ovarian cancer and 11 patients with sensitive recurrent ovarian cancer were included. The 5‐year OS for drug‐resistant recurrent ovarian cancer patients (44 ± 11.07 months, 95% CI: 231.24–53.66 months) was significantly lower than that of drug‐sensitive recurrent ovarian cancer patients (58 ± 3.97 months; 95% CI: 50.05–65.59 months; *p *=* *0.024) *TP53* was the most frequently mutated gene in both drug‐resistant (9/10, 90%) and drug‐sensitive recurrent ovarian cancers (10/11, 91%). *MYC* and *RB1* had the highest frequency of copy number variations (6/21, 29%) in recurrent ovarian cancers, followed by *PIK3CA* (3/21, 14%). *BRCA2* N372H polymorphism was found in 40% (4/10) of drug‐resistant recurrent ovarian cancer patients. The specificity, sensitivity, and consistency of *TP53* and *BRCA1* in circulating tumor‐free DNA and tumor tissue DNA were 100%, 73.7%, 76.2% and 100%, 75%, 95.24%, respectively.

**Conclusion:**

We uncovered extensive genetic alterations in recurrent ovarian cancer and drug‐resistant recurrent ovarian cancer exhibited unique genetic changes compared with recurrent ovarian cancer and drug‐sensitive recurrent ovarian cancer. We further showed that high‐throughput sequencing using liquid biopsy specimens could provide an effective, specific, and sensitive approach for detecting genetic alterations in ovarian cancer.

## 1 BACKGROUND

Ovarian cancer is a major cause of mortality from gynecologic cancers worldwide. Early ovarian cancer is often occult and difficult to detect. Due to lack of effective screening methods, approximately 70% of ovarian cancer patients are found at the late stage with extensive pelvic and abdominal metastasis. Despite the best therapeutic regimens available for patients with advanced ovarian cancer including surgical resection of curative intent and postoperative adjuvant chemoradiotherapy, most patients experience a relapse over time (Hanker et al., 2012).

Platinum‐based chemotherapy is the main postoperative adjuvant treatment of advanced ovarian cancer. However, 70%–80% ovarian cancer patients relapse after initial chemotherapy (Lin & Changchien, 2007). Primary or secondary resistance is the main cause for diminished effectiveness over time of platinum‐based chemotherapy, contributing to the dismal outcome of advanced ovarian cancer patients whose 5‐year survival rate is <30% (Schmalfeldt et al., 1995). Resistant recurrent ovarian cancer is defined as a tumor that relapses within 6 months of adjuvant chemotherapy. Currently, there is lack of cancer‐specific diagnostic biomarkers to monitor tumor evolution and predict the onset of resistance to chemotherapies.

Gene mutations and copy number variations have been identified in ovarian cancer, which contribute to oncogenesis, ovarian cancer progression, and acquired chemoresistance (Lu et al., 2017; Zhao, Sun, & Zhao, 2012). Delineation of gene mutations and copy number variations in ovarian cancer could not only provide insight into the interplay of mutated genes and their encoded proteins in driving tumorigenesis and tumor progression, but it could also lead to identification of diagnostic and prognostic biomarkers for primary and recurrent ovarian cancer. Furthermore, identification of biomarkers for chemoresistance of ovarian cancer could avoid continuing inefficacious therapies and prevent unnecessary toxicities.

Recently, circulating cell‐free DNA and circulating tumor cells have been found to offer a ready and convenient source for liquid biopsy (Xu et al., 2016); genetic alterations found in liquid biopsy specimens are identical to those in primary cancer tissues. Murtaza et al. (2013) showed that exome‐wide analysis of circulating tumor DNA could complement current invasive biopsy approaches to identify mutations associated with acquired drug resistance in advanced cancers including ovarian cancer. Circulating tumor DNA is a class of double stranded DNA fragments derived from tumor cells, ranging in size between 0.18 and 21 kb, mainly existing in the blood, synovial fluid and cerebrospinal fluid, and can be excreted through urine and feces. As a tumor dynamic marker, circulating tumor DNA has more advantages than traditional protein markers and imaging examination. Circulating tumor DNA has a short half‐life period (about 2 hr); so it can reflect changes in tumor within a few hours rather than a few weeks or months (Forshew et al., 2012). Therefore, circulating tumor DNA may show earlier tumoral changes than imaging studies or protein markers by several weeks and months (Dawson, Rosenfeld, & Caldas, 2013).

In the current study, by using high‐throughput sequencing technique, we sought to delineate genetic alterations in recurrent ovarian cancer patients and further compare genetic changes in drug‐resistant and ‐sensitive recurrent ovarian cancer patients. We also sought to study the specificity, sensitivity, and consistency of DNA biomarkers in liquid biopsy specimens and ovarian cancer tissue DNA.

## 2 PATIENTS AND METHODS

### 2.1 Tissue and fluid sample acquisition

Tumor tissue and fluid specimens were obtained from pathologically proven recurrent ovarian cancer patients who underwent surgical treatment at Shengjing Hospital of China Medical University, Shenyang, China, between January 2016 and January 2017. A patient was included in this study (1) if she had tumor recurrence after radical resection of primary ovarian cancer and postoperative adjuvant therapy; (2) if she was aged ≤70 years and the expected life expectancy was >3 months; (3) if her biopsy specimen of recurrent lesions was available for high‐throughput DNA sequencing analysis; (4) if she had normal cardiopulmonary function; (5) if her Karnofsky performance scale (KPS) score was >60; and (6) if she had no infections, bleeding and other complications. A recurrent ovarian cancer was considered drug‐resistant if ≤6 months elapsed between tumor recurrence and primary postoperative adjuvant chemotherapy, and drug‐ sensitive if >12 months elapsed between tumor recurrence and primary postoperative adjuvant chemotherapy.

The study protocol was approved by the local ethics committee at the authors’ affiliated institution. Patient consent was not required because of the retrospective nature of this study. Patient personal data were anonymized in the study.

### 2.2 Tissue and fluid sample preparation

Fresh surgical tumor tissue specimens were snap frozen and stored at −80°C. Nonfasting blood was collected in K_2_ EDTA tubes (Vacuette; Greiner, Germany) and then centrifuged at 700 *g* for 10 min and frozen immediately at −80°C before use. Midstream urine samples were collected in plastic urine collection cups and then transferred to 24‐ml polyethylene tubes and immediately frozen at −80°C. Ascites was drained by abdominal paracenthesis and 0.5–3 L ascitic fluid was centrifuged at 200 *g* for 5 min. The cell pellet was resuspended and then layered on 25 ml of Ficoll‐Paque (GE Healthcare, Uppsala, Sweden), and centrifuged at 510 *g* for 15 min with the brake off. The tumor cell‐rich fraction, in the interface between the medium fraction and the Ficoll fraction, was removed with a Pasteur pipette and transferred to a fresh tube. The cells were washed three times and viably frozen and stored at −80°C.

### 2.3 High‐throughput sequencing

Genomic DNA was extracted from tumor tissues and blood cells using the QIAamp FFPE Tissue Kit (Qiagen, Hilden, Germany) as instructed by the manufacturer. Peripheral blood samples were collected using cell‐free DNA BCT tube (Streck) and processed within 24 hr of sample collection. Plasma was separated by centrifugation at 820 *g* for 10 min. Circulating cell‐free DNA was isolated from 1 ml of plasma using the QIAamp Circulating Nucleic Acid kit (Qiagen) according to the manufacturer's instructions. DNA concentration was measured using a Qubit fluorometer (Life Technologies) and 100 ng DNA was sonicated for 30 s three times to obtain small DNA fragments 180–220 bp in size using a Diagenode™ Bioruptor™ Pico Ultrasonicator. DNA was stored at −20°C.

The DNA Library was constructed according to the instructions of the manufacturer. Genomic DNA (100 ng) was amplified using a commercially available kit according to the manufacturer's recommendations (Kapa). Each sample library was ligated with a specific barcode index according to the manufacturer's protocol (Kapa) and the DNA libraries were then pooled and captured using DNA‐capture probes (target 422 cancer‐related genes, Geneseeq). The samples are purified by AMPure XP beads, quantified by qPCR (Kapa) and sized on bioanalyzer 2100 (Agilent). Libraries were normalized to 2.5 nM and pooled. Deep Sequencing was performed on Illumina HiSeq 4000 using PE75 V1 Kit. Cluster generation and sequencing was performed according to the manufacturer's protocol.

### 2.4 Bioinformatic analysis

Base calling was performed using bcl2fastq v2.16.0.10 (Illumina, Inc.) to generate sequence reads in FASTQ format (Illumina 1.8+ encoding). Quality control (QC) was applied with Trimmomatic (Amarasinghe et al., 2014; Bolger, Lohse, & Usadel, 2014; Koboldt et al., 2012; Li & Durbin, 2009; McKenna et al., 2010; Robinson et al., 2011; Van der Auwera et al., 2013a). High‐quality reads were mapped to the human genome (hg19, GRCh37 Genome Reference Consortium Human Reference 37) using modified BWA aligner 0.7.12 (Li & Durbin, 2009) with BWA‐MEM algorithm and default parameters to create SAM files. Picard 1.119 (http://picard.sourceforge.net/) was used to convert SAM files to compressed BAM files which were then sorted according to chromosome coordinates. The Genome Analysis Toolkit(McKenna et al., 2010) (GATK, version 3.4‐0) was modified and used to locally realign the BAMs files at intervals with indel mismatches and recalibrate base quality scores of reads in BAM files (Van der Auwera et al., 2013a).

Single‐nucleotide variants (SNVs) and short insertions/deletions (indels) were identified using VarScan2 2.3.9(Koboldt et al., 2012) with minimum variant allele frequency threshold set at 0.01 and *p*‐value threshold for calling variants set at 0.05 to generate Variant Call Format (VCF) files. All SNVs/indels were annotated with ANNOVAR, and each SNV/indel was manually checked with the Integrative Genomics Viewer (Robinson et al., 2011) (IGV). Copy number variations (CNVs) were identified using ADTEx 1.0.4 (Amarasinghe et al., 2014).

### 2.5 Statistical analysis

The SPSS19.0 software (SPSS Inc., Chicago, IL) was used for statistical analysis and *p *<* *0.05 was considered statistically significant. A true positive mutation was defined as simultaneous detection of the same mutation in both circulating tumor DNA and the paired tissue and a true negative mutation was defined as failure to detect the same mutation in both circulating tumor DNA and the paired tissue. A false positive mutation was defined as detection of the same mutation in circulating tumor DNA but not in the paired tissue. A false negative mutation was defined as detection of the same mutation in the paired tissue but not in circulating tumor DNA. Specificity was calculated using the formula:

Specificity=true negative/(true negative+false positive)

Sensitivity was calculated using the formula:

Sensitivity=true positive/(true positive+false negative)

Consistency was calculated using the formula:

$$\begin{array}{c} \mathrm{Consistency}=(\mathrm{true}\mathrm{positive}+\mathrm{true}\mathrm{negative})/\mathrm{the}\mathrm{total}\mathrm{number}\mathrm{of}\mathrm{subjects} \end{array}$$

The tumor detection rate was defined the number of positive cancer tissue specimens or liquid biopsy samples for a specific cancer mutation divided by the total number of cancer tissue specimens or liquid biopsy samples multiplied by 100%. Quantitative data was analyzed using Student's *t* test and categorical data using chi‐squared test. Survival with 95% confidence interval (95% CI) was analyzed using the Kaplan–Meier method. The duration of overall survival (OS) was defined as the time from primary tumor radical resection to the date of death from any cause and the duration of progression‐free survival (PFS) was defined as the time from primary tumor radical resection to the earliest date of disease progression or death from any cause. COX regression univariate analysis and log‐rank test were used for analysis of prognostic factors.

## 3 RESULTS

### 3.1 Patient demographic and other baseline variables

Ten patients with pathologically proven drug‐resistant recurrent ovarian cancer and 11 patients with sensitive recurrent ovarian cancer were included in this study. Their age ranged from 39 to 70 years old (median 48 years old). Their demographic and baseline variables are shown in Table 1. The drug‐resistant recurrent ovarian cancer group included nine cases of poorly differentiated tumors and one case of high‐grade serous carcinoma. Three patients had the International Federation of Gynecology and Obstetrics (FIGO) stage II and seven patients had FIGO stage III recurrent ovarian cancer. The drug‐sensitive recurrent ovarian cancer group included eight cases of poorly differentiated tumors and three cases of high‐grade serous carcinoma (3/11). Three patients had FIGO stage II and eight patients had FIGO stage III recurrent ovarian cancer. Eight patients had poorly differentiated tumor (8/11), including low‐grade serous carcinoma in two cases, and high‐grade serous carcinoma in three in the drug‐sensitive recurrent ovarian cancer group and low‐grade serous carcinoma in six cases, and high‐grade serous carcinoma in one case in the drug‐resistant recurrent ovarian cancer group. The median time to tumor recurrence was 18 months (range 12–42 months) in the drug‐sensitive recurrent ovarian cancer group and 6 months (range 1–6 months) in the drug‐resistant recurrent ovarian cancer group (*p *=* *0.012). The two groups were incomparable in the demographic and other baseline variables.

**Table 1:** Patient demographic and baseline characteristics of recurrent ovarian cancer patients

Variables	Resistant ovarian cancer	Sensitive ovarian cancer	*p*
*N*	10	11	
Age			
Range	50–70	39–70	0.56
Median age	56	48	
FIGO stage			
II	3	3	0.63
III	7	8	0.63
Pathological type			
Low‐grade serious carcinoma	6	2	0.06
High‐grade serious carcinoma	1	3	0.33
Medium differentiated tumor	3	6	0.25

### 3.2 OS and PFS

**Figure 1:**
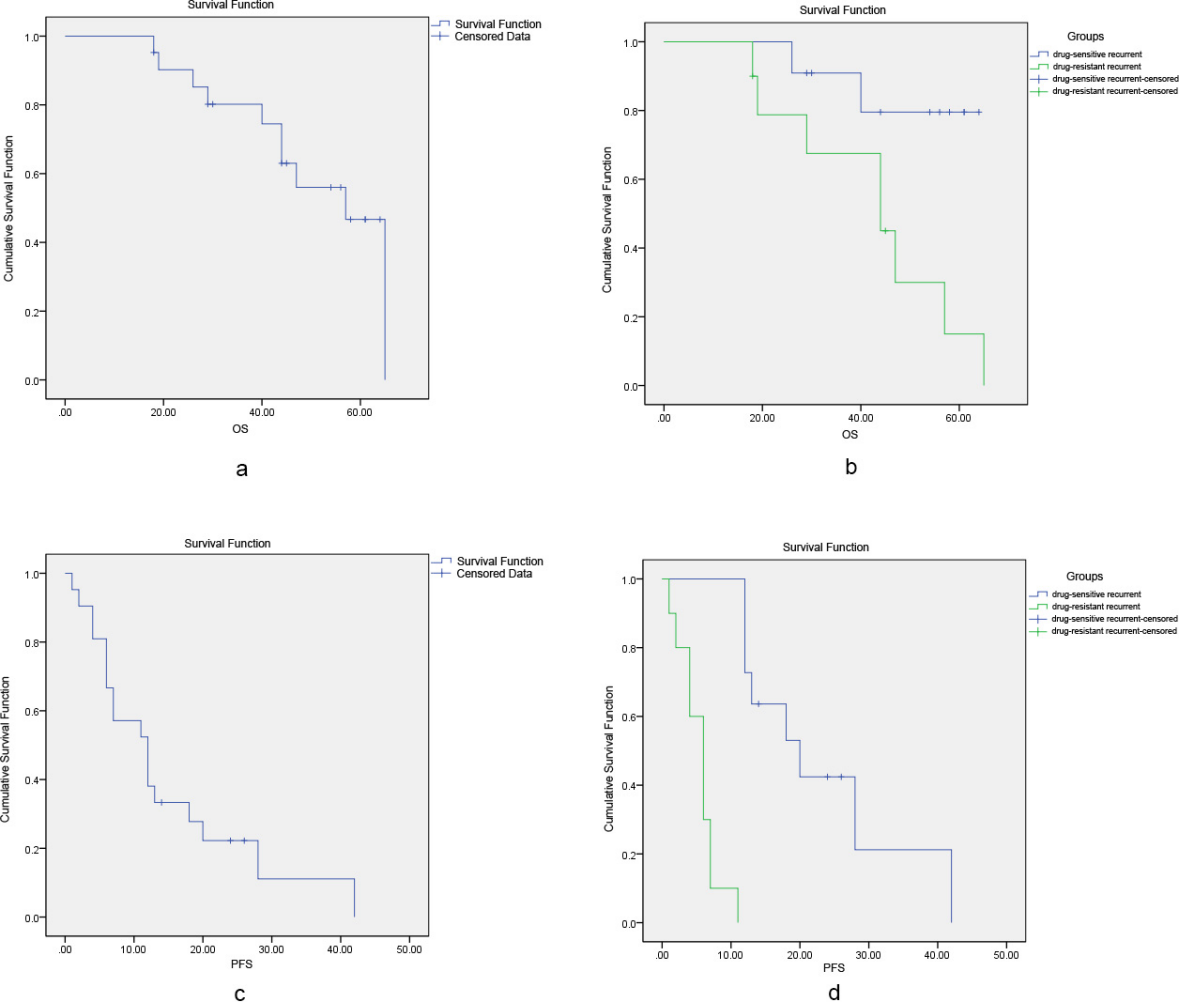
The Kaplan–Meier overall survival (OS) curve for the study cohort (a) and OS stratified by recurrent versus sensitive recurrent ovarian cancer (b). The Kaplan–Meier progression‐free survival (PFS) curve for the study cohort (c) and PFS stratified by recurrent versus sensitive recurrent ovarian cancer (d)

The mean 5‐year OS was 50.85 ± 3.97 months (95% CI: 43.07–58.63 months) for recurrent ovarian cancer patients (Figure 1a). The mean 5‐year OS was 42.45 ± 5.72 months (95% CI: 31.24–53.66 months) for drug‐resistant recurrent ovarian cancer patients, which was significantly lower than that of drug‐sensitive recurrent ovarian cancer patients (58 ± 3.97 months; 95% CI: 50.05–65.59 months; *p *=* *0.024; Figure 1b).

The mean 5‐year PFS was 14.79 ± 2.91 months (95% CI: 9.09–20.5 months) for recurrent ovarian cancer patients (Figure 1c). The mean 5‐year PFS was 5 ± 0.90 months (95% CI: 3.64–7.16 months) in drug‐resistant recurrent ovarian cancer patients, which was significantly lower than that of drug‐sensitive recurrent ovarian cancer patients (23.33 ± 4.1 months; 95% CI: 15.31–31.36 months; *p *<* *0.001; Figure 1d). COX regression univariate analysis and log‐rank test showed that the chemosensitivity of the recurrent tumor (sensitive *or* resistant) was an independent prognostic factor for OS (*p *=* *0.025, OR = 2.546, 95% CI: 0.041–6.958).

### 3.3 Cancer‐specific gene mutational profile of the study cohort

**Figure 2:**
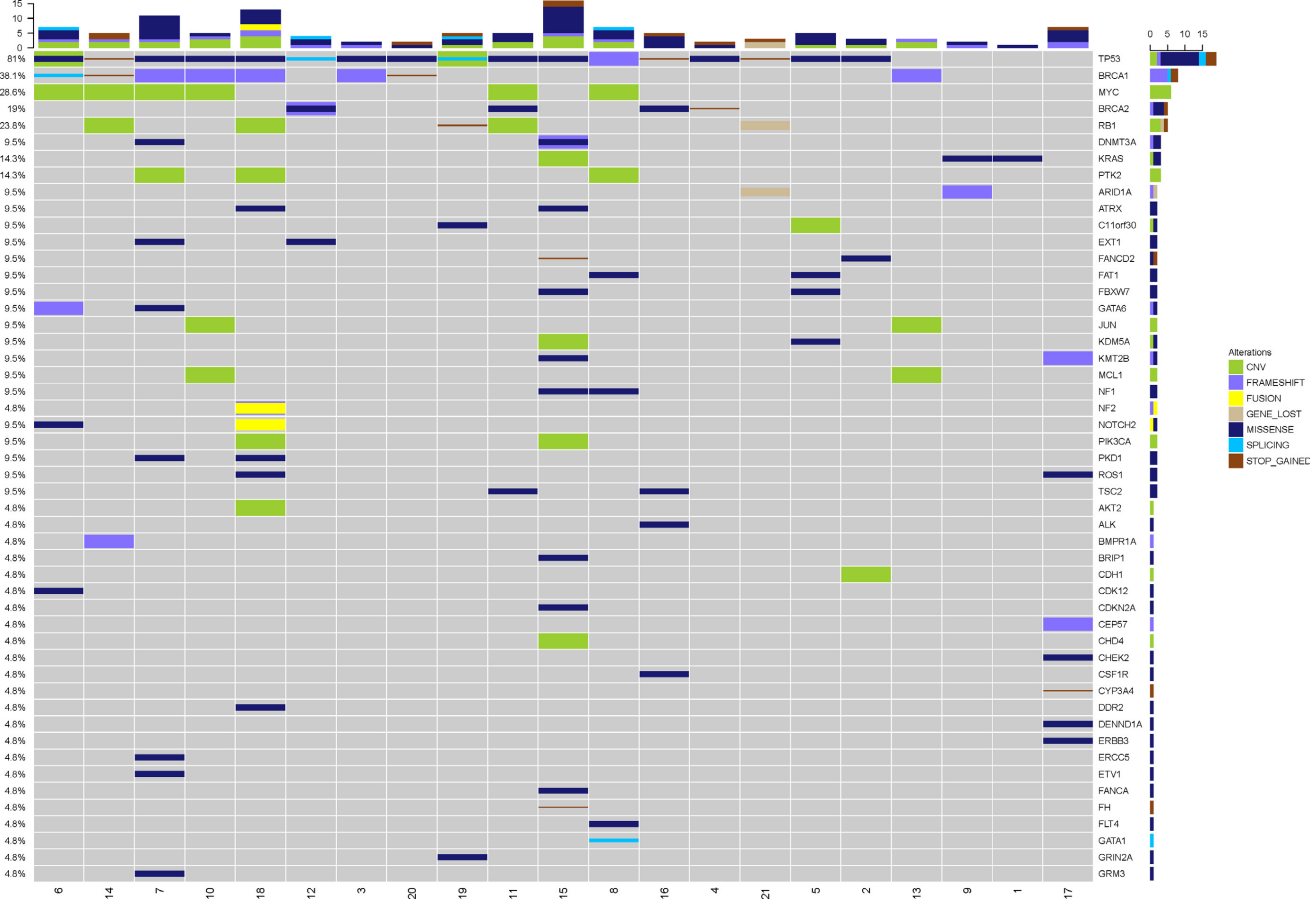
Summary of gene mutations in 21 recurrent ovarian cancer patients. Blue lines represent drug‐sensitive recurrent ovarian cancer and green lines indicate drug‐resistant ovarian cancer. Censored patients are indicated by a short vertical line

**Figure 3:**
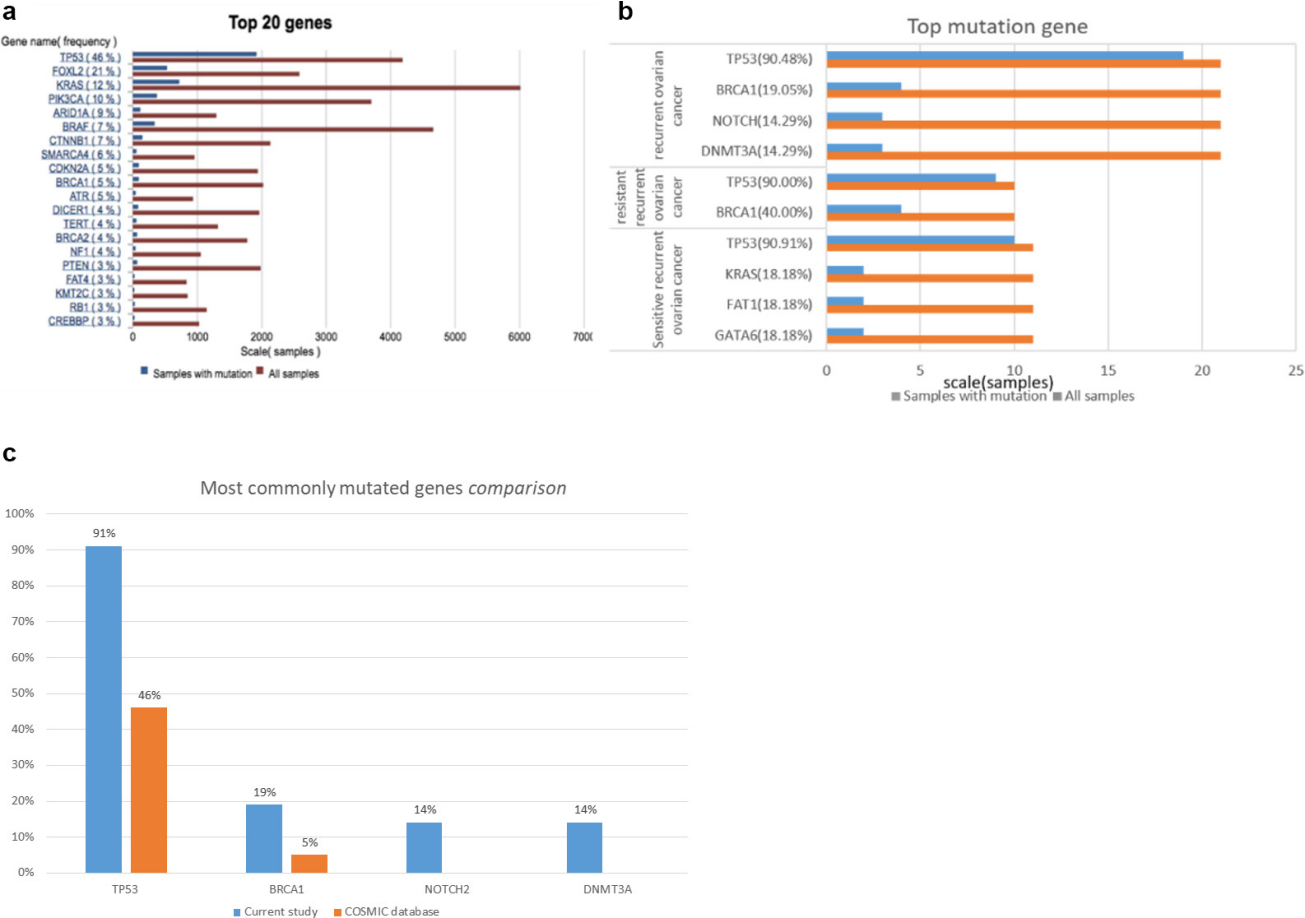
Tumor‐specific mutations. (a) The 20 most common mutations in ovary cancer in the cataloge of somatic mutations in cancer (COSMIC) database. (b). The most commonly mutated genes in recurrent ovarian cancer in this study, the ranking of mutant gene with higher frequency rate in drug‐resistant recurrent ovarian cancer and sensitive recurrent ovarian cancer. (c) Most commonly mutated genes comparison between current study and COSMIC database

The gene mutational profile by high‐throughput sequencing analysis of 21 patients with recurrent ovarian cancer is shown in Figure 2. *TP53, BRCA1, NOTCH2,* and *DNMT3A* are the four most commonly mutated genes in recurrent ovarian cancer compared to the COSMIC database (Figure 3c). *TP53* and *BRCA1* are the most frequently mutated genes in resistant recurrent ovarian cancer. By contrast, *TP53, KRAS, FAT1,* and *GATA6* are the most common ones in sensitive recurrent ovarian cancer (Figure 3b).

### 3.4 Copy number variation

*MYC* had the highest frequency of copy number variations (6/21, 29%) in recurrent ovarian cancer patients, followed by *RB1* (6/21, 29%) and *PIK3CA* (3/21, 14%). *TP53* (2/21, 10%), *PTK2* (2/21, 10%), *MCL1* (2/21, 10%), and *JUN* (2/21, 10%) also exhibited noticeably copy number variations. In drug‐resistant recurrent ovarian cancer patients, *RB1* had the highest frequency of copy number variations (4/10, 40%), which was followed by *MYC* (3/10, 30%). In drug‐sensitive recurrent ovarian cancer patients, *MYC* had the highest frequency of copy number variations (3/11, 27%), followed by *RB1* (2/11, 18%), and *PTK2* (2/21, 10%). *PIK3CA* (3/10, 30%), *TP53* (2/10, 20%), *MCL1* (2/10, 20%), and *JUN* (2/10, 20%) also showed noted copy number variations in drug‐resistant recurrent ovarian cancer and were specific for drug‐resistant recurrent ovarian cancer. *PTK2* copy number variation was unique to drug‐sensitive recurrent ovarian cancer.

### 3.5 Germline mutations

*BRCA2* N372H polymorphism was found in 40% (4/10) of drug‐resistant recurrent ovarian cancer patients, but was not detected in drug‐sensitive recurrent ovarian cancer patients.

### 3.6 Mutational characteristics of signaling pathways in recurrent ovarian cancer

Pathway analysis showed that the TP53 signaling pathway (mutation rate 80%), the DNA‐damage response signaling pathway (mutation rate 60%), the mTOR signaling pathway (mutation rate 60%), and the RTK signaling pathway (mutation rate 20%) were mutated in drug‐resistant recurrent ovarian cancer. The TP53 signaling pathway (mutation rate 73%), the DNA‐damage response signaling pathway (mutation rate 36%), and the RTK signaling pathway (mutation rate 37.3%) were also mutated in drug‐sensitive recurrent ovarian cancer. The TP53 signaling pathway (resistant: 80% vs. sensitive: 73%, *p *=* *0.55) and the DNA‐damage response signaling pathway (resistant: 60% vs. sensitive: 36%, *p *=* *0.26) had higher mutation rate in drug‐resistant recurrent ovarian cancer versus drug‐sensitive recurrent ovarian cancer. The RTK signaling pathway had numerically higher mutation rate in drug‐sensitive recurrent ovarian cancer versus drug‐resistant recurrent ovarian cancer (sensitive: 36% vs. resistant: 20%, *p *=* *0.55). But, there was no statistical difference between them. The mTOR signaling pathway was uniquely mutated in drug‐resistant recurrent ovarian cancer.

Mutational characteristics of the TP53 signaling pathway in drug‐resistant recurrent ovarian cancer are shown in Table 2. Common mutation sites included exon 5, 6, and 7 and missense mutation was the most common mutation type.

**Table 2:** Mutations in *TP53* in drug‐resistant recurrent ovarian cancer

No.	Site	Mutation type	Description	Expected consequence
1	Ex 2	Missense	c.G248T	p.S83I
2	Ex 3	Missense	c.T220G	p.F74V
3	Ex 5	Missense	c.G524A	p.R175H
4	Ex 5	Splicing	c.G376‐1C	–
5	Ex 5	Missense	c.G524A	p.R175H
6	Ex 6	Missense	c.569C>T	p.P190L
7	Ex 6	Missense	c.C637T	p.R213X
8	Ex 6	Missense	c.A659G	p.Y220C
9	Ex 7	Missense	c.G713T	p.C238F
10	Ex 7	Missense	c.T761G	p.I254S
11	Ex 7	Missense	c.G733A	p.G245S
12	Ex 7	Missense	c.G725T	p.C242F
13	Ex 8	Missense	c.809_834delTTGAGGTGCGTGTTTGTGCCTGTCCT	p.F270 fs
14	Ex 9	Missense	c.C949T	p.Q317X

### 3.7 Diagnostic characteristics of tumor‐free DNA and tumor tissue DNA

The tumor DNA detection rate in the plasma, ascites, and urine was 100%, 100%, and 86%, respectively, and the detection rate in tumor tissue was 100% (Table 3). The specificity, sensitivity, and consistency of *TP53* and *BRCA1* in circulating tumor‐free DNA and tumor tissue DNA were 100%, 73.7%, 76.2% and 100%, 75%, 95.24%, respectively (Table 4).

**Table 3:** Detection rate of tumor DNA in plasma, ascites, urine, and tissues in recurrent ovarian cancer patients

*N* = 5	Total cases	Tumor‐specific mutation	Tumor‐specific mutation
		Cases detected	Detection rate (%)
Plasma circulating tumor‐free DNA	21	21	100
Ascites	13	13	100
Urine	21	18	86
Tissue sample	21	21	100

**Table 4:** Sensitivity, specificity, and consistency of *TP53* in circulating tumor‐free DNA in plasma and tumor tissue DNA

*TP53*	Circulating tumor‐free DNA mutation			*BRCA1*	Circulating tumor‐free DNA mutation		
	Positive	Negative	Total		Positive	Negative	Total
Mutation in tumor tissue				Mutation in tumor tissue			
Positive	14	5	19	Positive	3	1	4
Negative	0	2	2	Negative	0	17	17
Total	14	7	21	Total	3	18	21

Consistency	Sensitivity	Specificity	Consistency	Sensitivity	Specificity
76.19%	73.68%	100%	95.24%	75%	100%

## 4 DISCUSSION

Cancer genome analysis has increasingly become a part of clinical care and development of next‐generation sequencing methodologies enables identification of mutated genes at scale. In this study, we showed that drug‐resistant ovarian cancer patients had significantly lower survival versus those with sensitive recurrent ovarian cancer, and by using high‐throughput sequencing technology, we unraveled extensive genetic alterations in recurrent ovarian cancer and found that drug‐resistant and ‐sensitive recurrent ovarian cancer, though sharing certain genetic alterations, exhibited distinct genetic changes.

Type I ovarian cancer, which includes low‐grade serous carcinoma, endometrioid carcinoma, clear cell carcinoma, and mucinous carcinoma, has been shown to exhibit *KRAS*,* BRAF*,* PI3KCA,* and *PTEN* mutations (Shih Ie & Kurman, 2004). Type II ovarian cancer, which includes high‐grade serous carcinoma, undifferentiated carcinoma and sarcoma, often has mutations in *BRCA1* or *BRCA2*, and *TP53*, which is consistent with our results (Table 1). Compared with 20 most common mutations in ovarian cancer in the COSMIC database, our cohort showed a markedly higher rate of mutation in *BRCA1* and *TP53*. *BRCA1* and *BRCA2* are canonical tumor suppressor genes; mutations in these two genes in women are associated with an increased lifetime risk of ovarian cancer (Antoniou et al., 2003). *BRCA1* was mutated in 19% of our patients, which, intriguingly, occurred exclusively in drug‐resistant recurrent ovarian cancer patients. BRCA1 is involved in many functions, such as repair of double‐strand DNA breaks, cell cycle regulation, gene transcription regulation, inhibition of cell proliferation, and apoptotic mediation. High BRCA1 expression in drug‐resistant ovarian cancer cells has been shown to enhance cellular DNA repair capability, making cells less susceptible to chemotherapeutic drugs and leading to drug resistance (Zhou, Smith, & Liu, 2003). We failed to detect *BRCA2* N372H polymorphism in drug‐sensitive recurrent ovarian cancer, but the *BRCA2* polymorphism was present in 40% of drug‐resistant recurrent ovarian cancer patients. Su et al. (2015) showed in a meta‐analysis that *BRCA2* N372H polymorphism was associated with a significantly increased risk of ovarian cancer. Here, we found that *BRCA2* N372H polymorphism was closely related to platinum resistance. What role, if any, these genetic alterations in *BRCA1* and *BRCA2* played in acquired chemoresistance of recurrent ovarian cancer remains to be investigated. Maxwell et al. (2017) showed that though most breast or ovarian tumors with germline *BRCA1*/*BRCA2* loss of function mutations respond to DNA damaging agents, some tumors do not show such a response.

*BRCA1*‐ and *BRCA2‐*mutated tumors acquire additional somatic mutations, as in *TP53*, to suppress induction of DNA damage cell‐cycle checkpoints. *TP53* is a frequently mutated gene in human cancers. It has been reported that 96% of serous ovarian tumors are prone to *TP53* mutations (Brachova, Thiel, & Leslie, 2013). In our study, *TP53* was mutated in approximately 90% of both resistant and sensitive recurrent ovarian cancer patients and pathway analysis revealed that the TP53 signaling pathway also showed high rates of mutation in both resistant (80%) and sensitive (73%) recurrent ovarian cancer. We found that the mutations in our patients were more concentrated in exons 5, 6, and 7. The R175H missense mutation of *TP53* identified in this study disrupts the binding domain of p53, thus inactivating p53 function. Another *TP53* mutation (c.G376‐1C) identified in the current study occurs in the splicing site in the upstream of exon 5 and leads to abnormal slicing of *TP53*, resulting in loss of p53 function. The P190L missense mutation in exon 6 of *TP53* has been reported in gastric cancer, prostate cancer, and other tumors. However, its clinical significance in ovarian cancer is unknown. The R213X mutation in exon 6 of *TP53* causes a premature termination of p53. Y220C, I254S, C242F, and G245S mutations in this study were previously described (Shpak, Goldberg, & Cowperthwaite, 2014) in lung cancer, esophageal squamous cell carcinoma, ovarian cancer, and colorectal carcinoma.

Itamochi et al. (2017) reported that aberrant RTK signaling was closely associated with prognosis of ovarian clear cell carcinoma and the 3‐year survival rate in patients with activated RTK signaling was higher than that of patients with inactivated RTK signaling (91% vs. 53%, hazards ratio 0.35 [95% CI: 0.13–0.94], *p *=* *0.0373). This study found comparable rates of mutation in the RTK signaling pathway in drug‐resistant and ‐sensitive recurrent ovarian cancer. We also found that the mTOR signaling pathway was uniquely mutated in drug‐resistant recurrent ovarian cancer and had a mutation rate of 60%. It was reported that the PI3K/AKT/mTOR pathway plays an important role in the progression of ovarian cancer (Dobbin & Landen, 2013). We found that in drug‐resistant recurrent ovarian cancer patients, *RB1* had the highest frequency of copy number variations (40%). Murtaza et al. (2013) studied plasma DNA in ovarian cancer patients who received cisplatin therapy and found a noticeable increase in *RB1* mutation in these patients. Loss of RB1 is associated with chemotherapy response (Knudsen & Knudsen, 2008).

We found the tumor DNA detection rate in the plasma and ascites reached 100%, and the specificity of *TP53* and *BRCA1* in circulating tumor‐free DNA was 100% and the sensitivity 73.7% for *TP53* and 75% for *BRCA1*, which are comparable to those of tumor tissue DNA (specificity 76.2% and sensitivity 100%). This is consistent with early findings showing that the sensitivity of liquid biopsy in patients with stage IV ovarian tumors is almost 100% (Santillan et al., 2007). The high sensitivity and specificity of cell‐free DNA in our study suggest that detection of DNA biomarkers from liquid biopsy by high‐throughput sequencing technology may allow for molecular heterogeneity assessment, dynamic monitoring, and monitoring the appearance of gene mutations related to drug resistance. Xu et al. (2016) reported a consistency of 76.2% for next‐generation sequencing technology in detecting gene mutation in tissue samples and cell‐free DNA of paired blood samples in advanced lung cancer patients. We found a consistency of 76.2% for *TP53* and 95.24% for *BRCA1* in circulating tumor‐free DNA and tumor tissue DNA, suggesting that cell‐free DNA and circulating tumor cells could offer a ready and convenient source for liquid biopsy for detection of biomarkers that could be used to monitor disease development and predict prognosis.

In conclusion, using high‐throughput sequencing, we uncovered extensive genetic alterations in recurrent ovarian cancer. Drug‐resistant recurrent ovarian cancer exhibited unique genetic changes compared with drug‐sensitive recurrent ovarian cancer. We further showed that high‐throughput sequencing using liquid biopsy specimens could provide an effective, specific, and sensitive approach for detecting genetic alterations in ovarian cancer.

## CONFLICT OF INTEREST

The authors declare there is no conflict of interest involved in this study.
